# Polyamidoamine (PAMAM) dendrimer conjugate specifically activates the A_3 _adenosine receptor to improve post-ischemic/reperfusion function in isolated mouse hearts

**DOI:** 10.1186/1471-2210-11-11

**Published:** 2011-10-31

**Authors:** Tina C Wan, Dilip K Tosh, Lili Du, Elizabeth T Gizewski, Kenneth A Jacobson, John A Auchampach

**Affiliations:** 1 Department of Pharmacology/Toxicology and the Cardiovascular Center, Medical College of Wisconsin, 8701 Watertown Plank Road, Milwaukee, WI 53226, USA; 2 Molecular Recognition Section, Laboratory of Bioorganic Chemistry, National Institute of Diabetes and Digestive and Kidney Diseases, National Institutes of Health, Bethesda, MD 20892-0810, USA

## Abstract

**Background:**

When stimulated by small molecular agonists, the A_3 _adenosine receptor (AR) mediates cardioprotective effects without inducing detrimental hemodynamic side effects. We have examined pharmacologically the protective properties of a multivalent dendrimeric conjugate of a nucleoside as a selective multivalent agonist for the mouse A_3_AR.

**Results:**

A PAMAM dendrimer fully substituted by click chemistry on its peripheral groups with 64 moieties of a nucleoside agonist was shown to be potent and selective in binding to the mouse A_3_AR and effective in cardioprotection in an isolated mouse heart model of ischemia/reperfusion (I/R) injury. This conjugate MRS5246 and a structurally related model compound MRS5233 displayed binding K_i _values of 0.04 and 3.94 nM, respectively, and were potent in *in vitro *functional assays to inhibit cAMP production. A methanocarba (bicyclo[3.1.0]hexane) ring system in place of ribose maintained a North conformation that is preferred at the A_3_AR. These analogues also contained a triazole linker along with *5'-N*-methyl-carboxamido and 2-alkynyl substitution, previously shown to be associated with species-independent A_3_AR selectivity. Both MRS5233 and MRS5246 (1 and 10 nM) were effective at increasing functional recovery of isolated mouse hearts after 20 min ischemia followed by 45 min reperfusion. A statistically significant greater improvement in the left ventricular developed pressure (LVDP) by MRS5246 compared to MRS5233 occurred when the hearts were observed throughout reperfusion. Unliganded PAMAM dendrimer alone did not have any effect on functional recovery of isolated perfused mouse hearts. 10 nM MRS5246 did not improve functional recovery after I/R in hearts from A_3_AR gene "knock-out" (A_3_KO) mice compared to control, indicating the effects of MRS5246 were A_3_AR-specific.

**Conclusions:**

Covalent conjugation to a versatile drug carrier enhanced the functional potency and selectivity at the mouse A_3_AR and maintained the cardioprotective properties. Thus, this large molecular weight conjugate is not prevented from extravasation through the coronary microvasculature.

## Background

Polyamidoamine (PAMAM) dendrimers can serve as biocompatible polymeric nanocarriers of bioactive molecules of interest. They are versatile in that their size, shape and placement of functional groups can be customized for various applications [1-5]. For example, dendrimers have been studied for targeted drug and gene delivery [4-8] or for use as experimental tools to carry markers to targeted cells that express unique receptors [9-11]. Additionally, the attachment of drugs to dendrimers can prevent their premature degradation or even enhance tissue selectivity [1]. Nucleoside derivatives that activate adenosine receptors (ARs) can also be attached to dendrimers, which can greatly improve potency or effectiveness of the nucleoside ligands [12-14].

**Figure 1:**
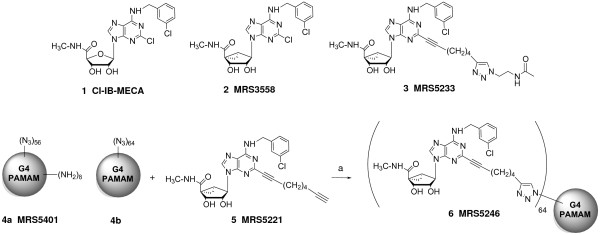
**Chemical structures of nucleoside and dendrimer derivatives**. MRS5221 **5 **is a *5'-N*-methyl-carboxamido adenosine analogue with a (N)-methanocarba ring system that served as a synthetic precursor for the conjugation to an azide-functionalized G4 PAMAM dendrimer **4b **to synthesize the GLiDe conjugate MRS5246 **6**. The monomer, MRS5233 **3 **contained a 2-alkynyl chain similar to MRS5221, but also contains a triazole linker group present in the PAMAM conjugate **6**. MRS5401 **4a **is a closely related unliganded PAMAM precursor molecule.

In the present study, an agonist of the A_3_AR conjugated to a PAMAM dendrimer was characterized pharmacologically and tested for protective effects in an isolated mouse heart model of ischemia/reperfusion (I/R) injury. The A_3_AR is one of four subtypes among a G protein-coupled receptor (GPCR) family (A_1_, A_2A_, A_2B _and A_3_) that responds to extracellular adenosine [15]. Adenosine levels are significantly increased during ischemic stress, thereby activating ARs near the site of injury [16-18]. Of the four subtypes, A_1 _and A_3_ARs present in the heart are well known to mediate the cardioprotective effects of adenosine [19-22]. However, unlike the A_1_AR, when the A_3_AR is activated by selective agonists, such as Cl-IB-MECA **1 **(Figure 1), it does not induce detrimental hemodynamic side effects, such as negative chronotropy, inotropy and dromotropy [20,23-25].

There is a medicinal chemical challenge in designing nucleosides that act as selective A_3_AR agonists across species. We have reported a class of A_3_AR agonists that are *5'-N*-methyl-carboxamido adenosine analogues containing a methanocarba (bicyclo[3.1.0]hexane) ring system in place of ribose to maintain a preferred North (N) conformation, but some of the analogues such as MRS3558 **2 **were shown to display >1,000-fold selectivity for the human (h) A_3_AR but not the mouse (m) and rat A_3_ARs [26,27]. This is because the (N)-methanocarba ring system is better tolerated at the A_1_AR in rodents than in humans, thereby, reducing selectivity for rodent A_3_ARs [26,27]. Thus, these agonists that are otherwise very selective in human are of limited use in rodent models. However, by varying substituents on the *N^6 ^*and C2 positions in the same structural series, species independent A_3_AR selectivity was restored, especially in analogues containing a 2-alkynyl (C≡C) group [27]. In the present study, we have applied the principle of enhancement of mA_3_AR selectivity of 2-alkynyl analogues to chain extended structures. Specifically, the dialkyne **5 **contains a second alkynyl group at the terminal position for conjugation by click chemistry (Cu(I)-catalyzed 1,3-dipolar cycloaddition reaction) to an azido derivatized PAMAM dendrimer **4b **to produce a GPCR-Ligand Dendrimer (GLiDe) conjugate. The click precursor **5 **also served as an intermediate for the model compound **3**. Both **3 **and the corresponding multivalent GLiDe conjugate MRS5246 **6 **were pharmacologically characterized at mouse ARs, and it was determined whether binding and functionality were affected by conjugation. Then, the cardioprotective effects of these monomeric and polymeric A_3_AR agonists were evaluated utilizing isolated mouse hearts in a Langendorff-perfusion system.

## Results

### Agonist structures and competition binding

We characterized the mAR binding of a GLiDe conjugate, MRS5246, and an unconjugated, structurally related monomer agonist, MRS5233 **3**. The GLiDe conjugate MRS5246 was synthesized, as reported [13,14], by covalently conjugating a precursor monomer, MRS5221 **5**, onto a fourth generation (G4) fully-substituted azido PAMAM dendrimer precursor **4b **(Figure 1). The monomeric compound, MRS5233 **3**, mimicked the structure of the modification of MRS5221 **5 **after conjugation to the dendrimer. Thus, the Cu(I)-catalyzed click reaction forms a triazole from the combination of an azido group (on the PAMAM) and a terminal alkyne (on the nucleoside).

**Figure 2:**
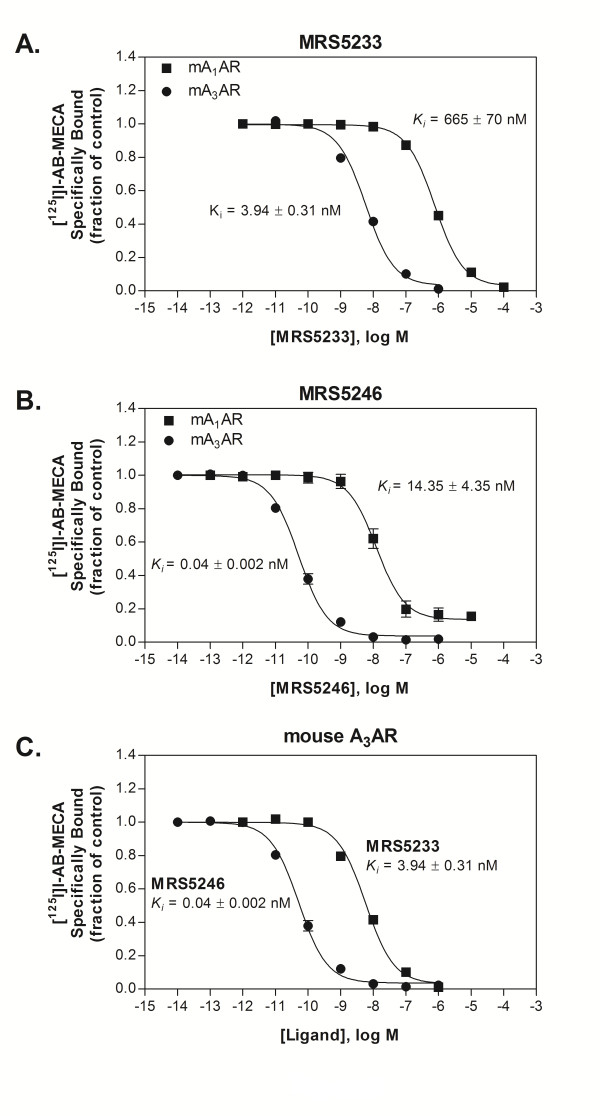
**Competition binding assays**. Increasing concentrations of MRS5246 or MRS5322 were added to compete for binding with [^125^I]I-AB-MECA to determine binding affinity, expressed as K_i _or K_iapp_, to recombinant mARs on HEK293 cell membranes. A & B, Both MRS5246 and MRS5322 bind to the mA_3_AR with much higher affinity than to the mA_1_AR. C, binding curves from mA_3_ARs to compare and show MRS5246 binds with much higher affinity than MRS5322 at the mA_3_AR.

From competition radioligand binding assays, it was determined that the GLiDe conjugate, MRS5246, and the monomer, MRS5233, both bind with significantly higher affinity to the mA_3_AR than to the mA_1_AR by 359- and 169-fold, respectively (Table 1 & Figure 2A and 2B). When comparing the selectivity of these agonists at the mA_3_AR, the GLiDe conjugate MRS5246 showed a 98.5-fold higher affinity than the monomer MRS5233 (Table 1, Figure 2C).

**Table 1:** Binding affinities calculated from radiologand ([^125^I]I-AB-MECA) binding experiments.

Agonist	**mA_3_AR**, K_i _or K_iapp _(nM)	**mA_1_AR**, K_i _or K_iapp _(nM)	Selectivity (A_1 _/A_3_)
**MRS5246**	0.04 ± 0.002	14.35 ± 4.35	359-fold

**MRS5233**	3.94 ± 0.31	665 ± 70	169-fold

	98.5-fold increase (MRS5233/MRS5246)	46.3-fold increase (MRS5233/MRS5246)	

### cAMP functional assays

**Figure 3:**
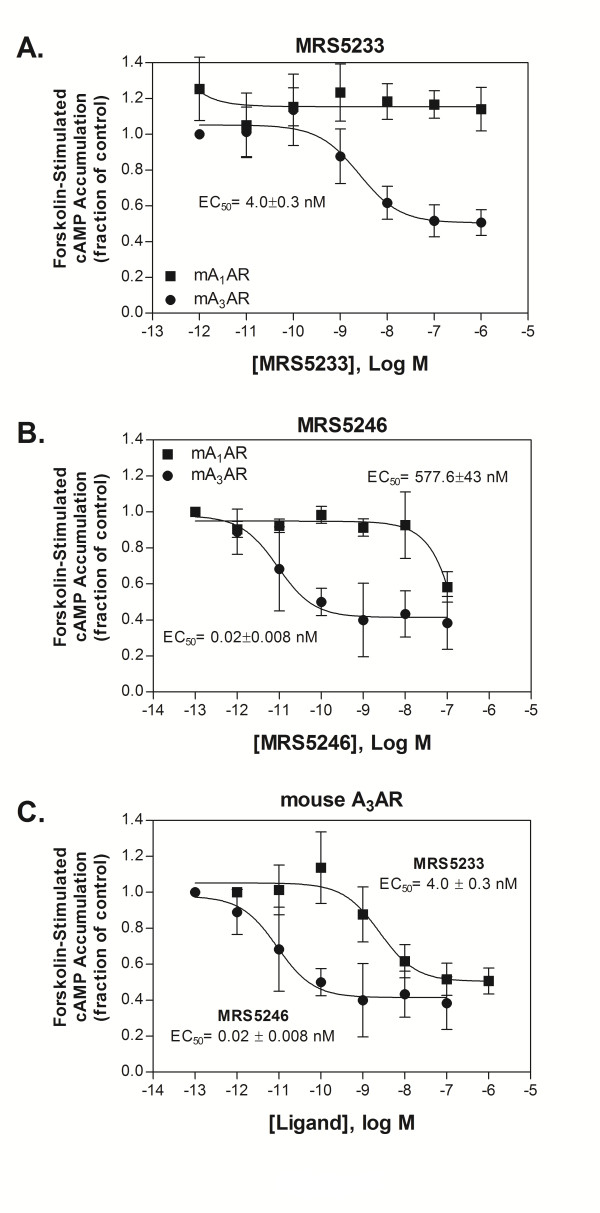
**cAMP functional assays**. Increasing concentrations of MRS5246 or MRS5322 were added to activate mA_1 _or mA_3_ARs to inhibit forskolin-stimulated cAMP production. A & B, both compounds are much more potent at the mA_3_AR than at the mA_1_AR. C, curves from mA_3_ARs to compare and show MRS5246 was much more potent than MRS5233 at the mA_3_AR.

cAMP assays in HEK293 cells overexpressing recombinant mARs were utilized to determine the potency, expressed as IC_50, _of MRS5246 and MRS5233. Both MRS5246 and MRS5233 were full agonists and highly selective for the mA_3_AR in comparison to the mA_1_AR (Figure 3A and 3B). In fact, MRS5233 at 100 μM was inactive at the mA_1_AR. When the conjugate MRS5246 was compared to the monomer MRS5233 in activating the A_3_AR, the conjugate was 200-fold more potent (0.02 nM vs. 4.0 nM) (Figure 3C).

### Isolated mouse heart I/R experiments

MRS5246 and MRS5233 were tested for effects on functional improvement in isolated mouse hearts subjected to global ischemia and reperfusion. The agonists were infused into the coronary circulation of the hearts for 10 min immediately before 20 min of global ischemia. The functional recovery parameters (left ventricular developed pressure [LVDP], rate of contraction [dP/dt_max_], rate of relaxation [dP/dt_min_], and coronary flow [CF; mL/min/g]) of the hearts were assessed throughout reperfusion and after 45 min of reperfusion and compared to those of control hearts.

**Figure 4:**
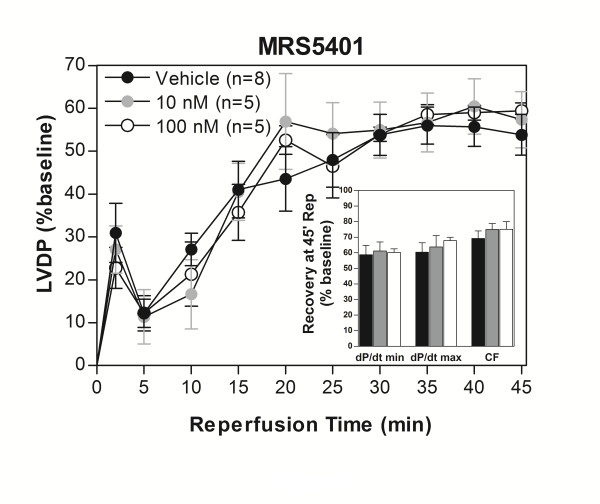
**PAMAM control dendrimer, MRS5401, alone did not influence functional recovery of isolated mouse hearts subjected to 20 min ischemia/45 min reperfusion up to a concentration of 100 nM**. The graph shows recovery of LVDP during reperfusion as a percent of paced baseline. The inset shows the percent recovery of the hearts at 45 min reperfusion for the rates of relaxation, contraction and coronary flow. There were no differences compared to control hearts not exposed to MRS5401.

**Figure 5:**
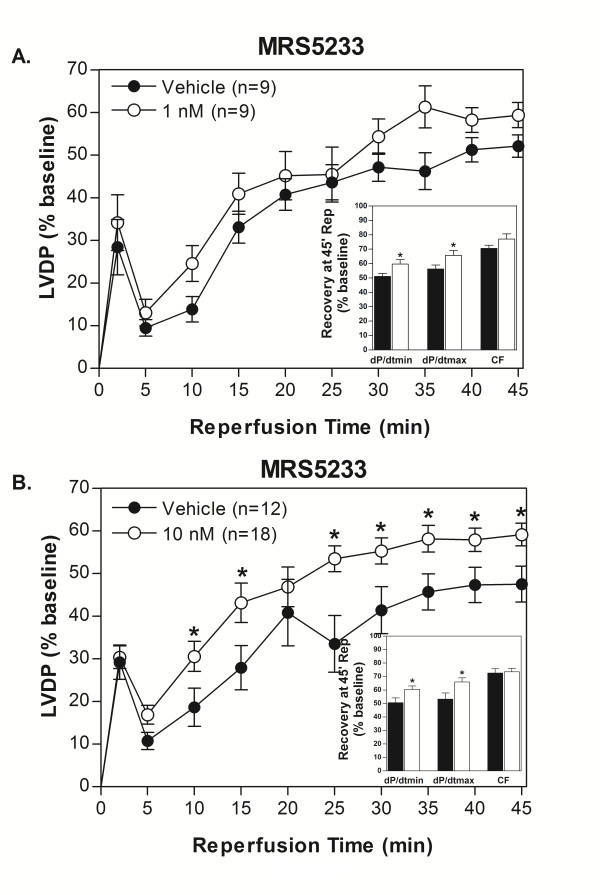
**The monomeric analogue, MRS5233, was effective at increasing functional recovery of isolated mouse hearts after 20 min ischemia/45 min reperfusion compared to control at 1 (A) and 10 nM (B)**. 1 nM MRS5233 showed a trend in improving LVDP over control during reperfusion, while there was significant improvement with 10 nM MRS5233. The insets show that the recovery of rates of relaxation and contraction at 45 min reperfusion was significantly better than that of control hearts at both concentrations.

**Figure 6:**
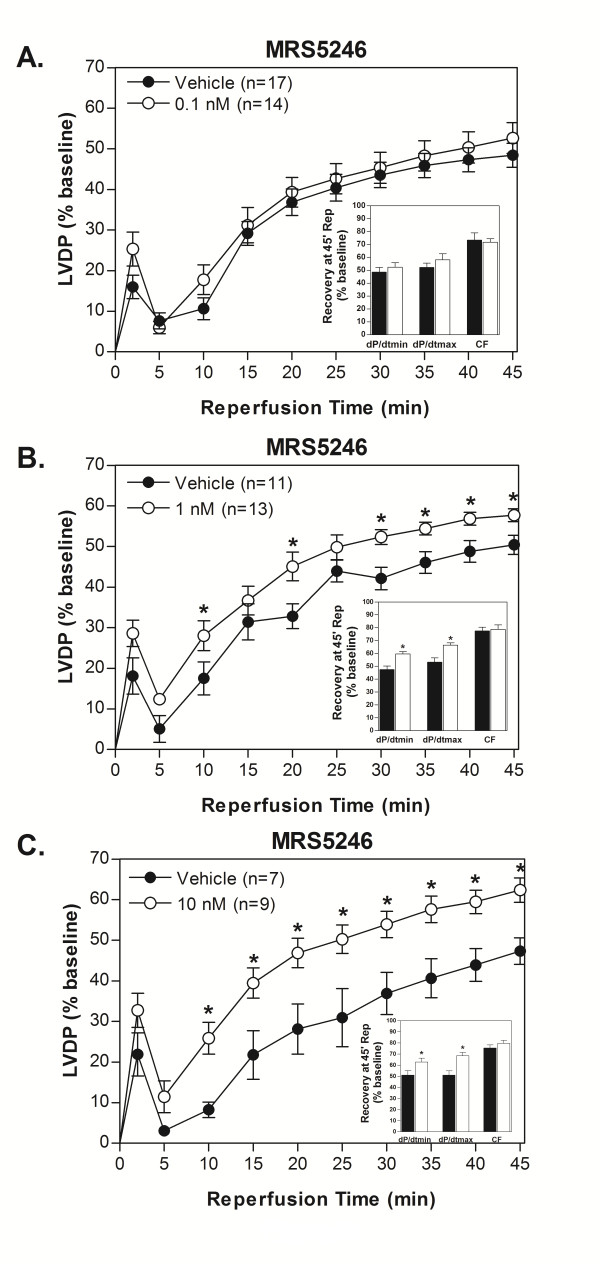
**The GLiDe conjugate, MRS5246, was effective at increasing functional recovery of isolated mouse hearts after 20 min ischemia/45 min reperfusion compared to control at 0.1 (A), 1 (B), and 10 nM (C)**. Both 1 and 10 nM concentrations of MRS5246 evoked significant improvement of LVDP over control during reperfusion. The insets show that there was also significantly better recovery of rates of relaxation and contraction at 45 min reperfusion than that of control hearts at both concentrations.

**Figure 7:**
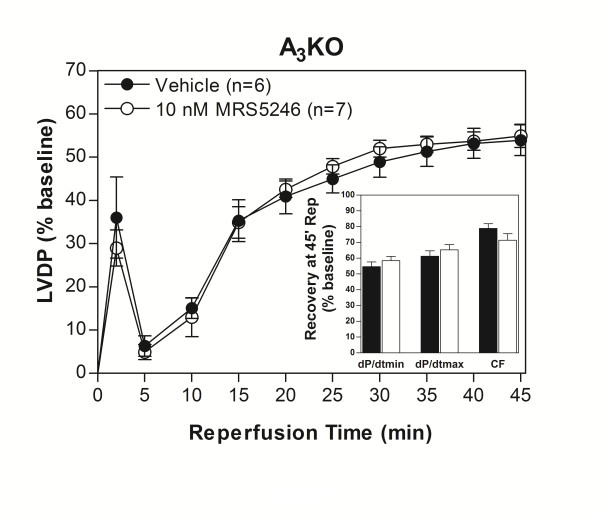
**Hearts from A_3_KO mice exposed to 10 nM MRS5246 did not show improved functional recovery after I/R compared to control indicating the effects of MRS5246 were mA_3_AR-specific**.

First, it was confirmed that the nearly fully azide-derivatized PAMAM dendrimer precursor not bearing any nucleoside moieties, MRS5401 **4a **(Figure 1), did not invoke any functional effects. There was no improvement of functional recovery following I/R after the hearts were exposed to ≤100 nM MRS5401 (Figure 4). However, the monomeric agonist, MRS5233, and GLiDe conjugate, MRS5246, evoked improved functional recovery compared to control in a concentration-dependent manner (Figures 5 and 6). MRS5246 provided marginally better improvement compared to MRS5233 at both 1 and 10 nM. MRS5246 at the lower concentration of 1 nM produced a statistically significant improvement in the recovery of LVDP throughout reperfusion whereas an equivalent concentration of MRS5233 did not. Thus, MRS5246 exhibited greater potency at providing ischemic protection. 10 nM MRS5246 was also infused into hearts from A_3_KO mice and subjected to I/R. There was no effect on functional improvement in these hearts compared to control hearts (Figure 7).

## Discussion

PAMAM dendrimers are biocompatible polymers with extensive and advantageous applications in drug delivery *in vivo*. Previous data have shown GLiDe conjugates and other nanoconjugates to acquire beneficial pharmacological characteristics, such as increased receptor affinities, increased potency and/or increased selectivity in comparison to monomeric ligands [1-5,9-14]. These nanocarriers may also provide pharmacokinetic and pharmacodynamic advantages, such as impeded metabolic degradation or tissue selectivity [1-5,9-14]. In our case, the drug is not intended for cleavage from the carrier at the site of action. The extracellular intact conjugate holds the relevant biological activity and can benefit from the versatility of the PAMAM dendrimers for further derivatization or structural modification as needed for the biological application.

For the present studies, an adenosine analogue (MRS5233) selective for the hA_3_AR, but modified to possess improved selectivity for the A_3_AR in mouse, was characterized in mouse model experiments [27]. This compound was also investigated as a mA_3_AR agonist pharmacophore for conjugation to a G4 PAMAM dendrimer for the synthesis of the GLiDe conjugate, MRS5246.

It was determined that both agonists, MRS5246 and MRS5233, bind with high affinity to the mA_3_AR with K_i _values of 0.04 and 3.94 nM, respectively. In comparison to other AR subtypes, they are highly selective for the mA_3_AR. This includes high selectivity over the A_1_AR, the subtype to which these analogues might be more likely to bind compared to A_2A _or A_2B_ARs. It was further determined that MRS5233 and MRS5246 are highly potent in *in vitro *functional assays to inhibit cAMP production via A_3_AR-induced activation of G_i _proteins compared to that of the A_1_AR. MRS5246 showed an IC_50 _of 0.02 nM at the mA_3_AR compared to 577.6 nM at the mA_1_AR (ratio of 28,880), while MRS5233 resulted in an IC_50 _of 4.0 nM at the mA_3_AR and lacked inhibition at the A_1_AR up to 0.1 mM. Therefore, these compounds promised to be excellent candidates for the study of cardioprotection by A_3_AR activation in mouse models.

In comparing the activities of the two compounds, MRS5246 possessed a much higher affinity to the mA_3_AR compared to the monomer, MRS5233, by 98.5-fold. The conjugated MRS5246 was also 200-fold more potent A_3_AR agonist than the monomer, MRS5233, in an *in vitro *cAMP assay. These findings are comparable to previous findings with other GLiDe conjugates, which significantly improved affinity and potency over the unconjugated monomeric agonists [13,14,28]. The K_i _values of these agonists in binding to hARs have been reported and indicate that **3**, **5**, and **6 **are all A_3_AR-selective [13,14,27]. For example, the triazole derivative **3 **displayed K_i _values of 22.3 ± 1.6 nM and 2440 ± 320 nM at the hA_3_AR and hA_2A_AR, respectively, and at 10 μM inhibited only 12 ± 4% of the radioligand binding at the hA_1_AR [13,14]. Thus, the model compound **3 **binds to the mA_3_AR with 5.7-fold greater affinity than to the hA_3_AR. The conjugate **6 **binds to the mA_3_AR with 3.5-fold greater affinity than to the hA_3_AR. In comparison to the A_1_AR, the A_3_AR selectivity of **6 **is slightly greater in the mouse (359-fold) than human (161-fold).

The binding and *in vitro *cAMP assays were performed under unrestricted solution conditions, with no intervening biological barriers such as would be encountered *in vivo*. There is limited hindrance in these experiments for the PAMAM dendrimers, which range in size of around ~45 Å in diameter for a G4 dendrimer [29]. This size estimate does not include the addition of the covalently conjugated agonist moieties, which would further increase the diameter. However, *in vivo*, the GLiDe conjugates must reach the myocardial targets by extravasation from the coronary microvasculature. It has been determined that the extravasation process of PAMAMs alone is dependent on the size and maybe even charge of the compounds [29]. These authors found that with each size-increasing generation of PAMAM dendrimers, up to the G4, there was a weight- and size-dependent decrease in the extravasation of the dendrimers. There was a steady decrease in the rate of extravasation from the G1 to G3 dendrimers, but an especially high decrease between G3 and G4 [29].

However, in light of these intuitive results that greater sizes offer hindrances, the Langendorff isolated heart experiments showed that the GLiDe conjugate, MRS5246, was just as, maybe even more, effective than the monomeric agonist in generating cardioprotection after I/R. MRS5246 and MRS5233 were effective in a concentration-dependent manner, where both showed significant protection starting at one nanomolar, and the effects were greater at 10 nM. The percentage of improvements after 45 min of reperfusion was marginally greater for the GLiDe conjugate at these concentrations. However, the multivalent conjugate MRS5246 (1 nM) was more effective than the monomer MRS5233 in improving LVDP over control when observing the hearts throughout reperfusion. Since we did not obtain a direct measure of cell death, it should be noted that we do not know whether the improvements in functional recovery with agonist treatment observed in the isolated mouse heart studies resulted from protection against reversible injury (i.e., myocardial "stunning") and/or necrotic cell injury (i.e., infarction).

## Conclusions

Conjugation of an A_3_AR-specific agonist to a PAMAM dendrimer indeed greatly improved its *in vitro *affinity and potency compared to the monomer itself. However, despite the greatly increased size and bulkiness of the GLiDe conjugate, it retained its potent cardioprotective effects in mouse isolated perfused hearts, and it was more effective than its smaller monomeric counterpart. This implied that the barrier for extravasation from the coronary microvasculature did not prevent passage of at least some of the polymeric conjugate.

These findings provide assurance that GLiDe conjugates warrant further study *in vivo *of their very important advantages over the unconjugated ligands. Because ARs are expressed throughout the body and produce different effects, these specific mA_3_AR dendrimers can be utilized to investigate methods to specifically target them to the myocardium, perhaps on the basis of an antibody complex. It has been determined that there is enhanced stability of ligands when they are conjugated to PAMAM dendrimers [30]. Protection of A_3_AR agonists against early degradation in the form of GLiDe conjugates is essential, especially considering the findings that A_3_AR agonists may be cardioprotective, but some are very short-lived. It is also becoming evident that GPCRs, including ARs, may function more efficiently as homo- or heterodimers [31-33]. These dendrimers, which provide ways to control the dimensions or spacing between the functional groups, will be important for studying ARs as oligomers. Additionally, other GPCR-binding ligands, such as opioids, also provide cardioprotection, in addition to findings that adenosine and opioid receptors can dimerize [34]. These dendrimeric carriers can be utilized to carry multiple agents to targeted tissues as enhanced cardioprotectants.

## Methods

### Chemical synthesis and reagents

Adenosine agonists **3**, **5**, and **6 **were synthesized as reported [13,14]. Adenosine agonists were first dissolved in DMSO and stored at -20 °C as stock solutions. PSB603 was purchased from Tocris Biosciences (Ellisville, MO), CVT-6883 was obtained from ChemieTek (Indianapolis, IN), and adenosine deaminase (ADA) was from Roche Applied Science (Indianapolis, IN). All other chemicals were from from Sigma-Aldrich (St. Louis, MO).

### Radioligand competition assays

The competition assays were performed as previously published [35]. Briefly, to prepare the membranes, HEK293 cells stably expressing recombinant mARs were harvested and homogenized in 10 mM Na^+^-HEPES buffer (pH 7.4) with 10 mM EDTA and 0.1 mM benzamidine. The cell lysates were centrifuged (35,000 × g for 25 min; 4°C), and the pellets were resuspended in 10 mM Na^+^-HEPES buffer (pH 7.4) with 1 mM EDTA and 0.1 mM benzamidine. The suspensions were homogenized and centrifuged again (35,000 × g for 25 min; 4°C). The resultant pellets were re-suspended in buffer containing 10% sucrose and stored at -80°C until use.

For the radioligand binding assays, 50 μg cell membranes (100 μL) were incubated in 10 mM Na^+^-HEPES buffer (pH 7.4) with 5 mM MgCl_2_, 5 U/mL ADA, and [^125^I]I-AB-MECA as radioligand. The competing ligands of interest were added at various concentration ranges. The IC_50 _values were calculated using nonlinear regression analysis by fitting the data to the following equation:

$$\text{binding}=\text{nonspecific binding}+\left[\left(\text{total binding}-\text{nonspecific binding}\right)∕\left(\text{1}+\text{1}0^{\text{x}-\text{logIC5}0}\right)\right]$$

Inhibition constant (K_i_) values were calculated using the Cheng-Prusoff equation [36]. The concentration of MRS5246 reflected the concentration of the dendrimeric complex, not the individual ligand moieties. Therefore, all binding K_i _values of this dendrimer are to be considered as K_iapp _values.

### cAMP functional assays

The assays of cAMP (3',5'-cyclic adenosine monophosphate) were performed utilizing a modified protocol published by Nordstedt and colleagues [37], whereby cAMP content was determined by displacement of [^3^H]cAMP binding to regulatory subunits of protein kinase A in bovine adrenal extracts. HEK293 cells heterologously expressing the mA_3_AR were prepared by plating 2.5 × 10^5 ^cells per well in 24-well plates and cultured overnight in complete media (DMEM with 10% fetal bovine serum/PenStrep/0.4 mg/mL G418) at 37°C. The cells were washed with DMEM/25 mM HEPES (pH 7.4) followed by incubation in DMEM/25 mM HEPES containing 20 μM Ro 20-1274 (phosphodiesterase inhibitor), 1 U/mL ADA and a combination of two selective A_2B_AR antagonists (300 nM CVT-6883 and 300 nM PSB603) at 37°C for 15 min. Forskolin (20 μM) to induce cAMP production and the test compounds of interest were then simultaneously added. A concentration range of ligands or dendrimer conjugates was tested in triplicate to inhibit cAMP production via activation of A_1 _or A_3 _ARs. After incubation at 37°C for 15 min, the media was aspirated, and the cells were lysed with 200 μL ice cold 0.1 M HCl on a shaker at 4°C for 30 min. The lysates were collected and cAMP levels in the acid extract were determined by competitive binding assays as follows. Each lysate sample and cAMP standard were diluted in 0.1 M HCl and subsequently transferred to polypropylene tubes (100 μL). [^3^H]cAMP (12,000 cpm/100 μL water/tube) and protein extract from bovine adrenal tissue were then added (240 μg adrenal protein/tube at a concentration of 0.6 μg/μL, in the binding protein buffer: 100 mM Tris-HCl, 250 mM NaCl, 10 mM EDTA, 0.1% 2-mercaptoethanol, pH 8.0), with the extract isolated from bovine adrenal glands as described by Nordstedt, *et al*. [37] to provide the cAMP binding substrate. The samples were incubated at 4°C for 2.5 hours and harvested through GF/C glass microfiber filters using a cell harvester (Brandel, Gaithersburg, MD) and counted in scintillation cocktail. The results were interpolated from standard curves obtained from known concentrations of cAMP standards.

### Animals

All experiments were performed with male mice (10-12 weeks of age, weighing ~25-30 g). Wild-type C57BL/6J mice were purchased from Jackson Laboratories (Bar Harbor, Me). A_3_KO mice were generated by embryonic stem cell targeting and genotyped by Southern blotting [38]. A_3_KO mice used had been transferred to the C57BL/6 genetic background by backcrossing greater than 12 generations. All experiments involving animals were approved by the Institutional Animal Care and Use Committee at the Medical College of Wisconsin and complied with the procedures established by the National Institutes of Health *Guide for the Care and Use of Laboratory Animals*.

### Langendorff-Perfused Isolated Mouse Heart Model of I/R

Isolated mouse heart experiments were performed as described previously [20,23]. In brief, hearts were isolated from C57Bl/6J male mice and cannulated via the aorta and perfused in a retrograde fashion by Krebs-Henseleit buffer. Pressure measurements were obtained via a fluid-filled balloon inserted into the left ventricle via the mitral valve and connected to a pressure transducer. The hearts were immersed in warmed perfusion buffer to maintain the hearts at 37°C, and the balloons were inflated to achieve constant end-diastolic pressures of 5 to 10 mm Hg.

The hearts were perfused for 20 min to achieve stabilization and then paced at 420 beats/min for baseline functional measurements. Global ischemia was achieved by closing an in-line stopcock to stop perfusion for 20 min followed by 45 min of reperfusion by re-opening the stopcock. To examine the effect of the ligands and dendrimer conjugates on functional recovery, the hearts were perfused with buffer containing the indicated concentrations of compounds for 10 min immediately before ischemia.

### Data Analysis

All data are reported as means ± S.E.M. Functional recovery data in the isolated heart studies were analyzed by two-way repeated measures ANOVA. If global tests showed a main effect, post-hoc contrasts between treatments were performed with Student's *t *test for unpaired data with the Bonferroni correction. Left ventricular functional recoveries at 45 min of reperfusion were compared using one-way analysis of variance followed by Student's *t *test with the Bonferroni correction or an unpaired Student's *t *test, as appropriate. *P *< 0.05 was statistically significant.

## Abbreviations

ADA: adenosine deaminase; AR: adenosine receptor; cAMP: 3',5'-cyclic adenosine monophosphate; CF: coronary flow; CVT-6883: 3-ethyl-1-propyl-8-(1-(3- trifluoromethylbenzyl)-1H-pyrazol-4-yl)-3,7-dihydropurine-2,6-dione; DMSO: dimethylsulfoxide; DMEM: Dulbecco's modified Eagle's medium; EDTA: ethylenediaminetetraacetic acid; *K*_iapp_, apparent inhibition constant; GLiDe: GPCR-ligand dendrimer; GPCR: G protein-coupled receptor; Cl-IB-MECA: 2-chloro-*N*^6^-(3-iodobenzyl)-5'-*N*-methylcarboxamidoadenosine; HEK: human embryonic kidney; HEPES: 4-(2-hydroxyethyl)-1-piperazineethanesulfonic acid; [^125^I]I-AB-MECA: [^125^I]4-amino-3-iodobenzyl-5'-*N*-methylcarboxamidoadenosine; LVDP: left ventricular developed pressure; PAMAM: poly(amidoamine); PSB603: 8-[4-[4-(4-chlorophenzyl)piperazide-1-sulfonyl)phenyl]]-1-propylxanthine.

## Authors' contributions

TCW planned and performed the animal experiments and wrote the manuscript. LD and ETG performed pharmacological experiments and contributed to assembly of the manuscript. DLT performed chemical synthesis and contributed to the manuscript. KAJ and JAA conceived of the study, planned the experimental strategy, and wrote the manuscript. All authors read and approved the final manuscript.

## Authors' information

KAJ is a Senior Investigator at NIDDK, NIH and is a medicinal chemist. JAA is a Professor of Pharmacology/Toxicology at the Medical College of Wisconsin and is a pharmacologist. The research of both groups is focusing on the roles of adenosine receptors.
